# Dark under the Lamp: Neglected Biological Pollutants in the Environment Are Closely Linked to Lung Cancer

**DOI:** 10.3390/ijms25063081

**Published:** 2024-03-07

**Authors:** Dongjie Wang, Ben Chung-Lap Chan, Bitian Zhang, Katie Ching-Yau Wong, Lea Ling-Yu Kan, Chun-Kwok Wong

**Affiliations:** 1 Institute of Chinese Medicine, State Key Laboratory of Research on Bioactivities and Clinical Applications of Medicinal Plants, The Chinese University of Hong Kong, Hong Kong, China; dongjiewang@cuhk.edu.hk (D.W.); benchan99@cuhk.edu.hk (B.C.-L.C.); bitianzhang@link.cuhk.edu.hk (B.Z.); lea-kan@cuhk.edu.hk (L.L.-Y.K.); 2 Department of Chemical Pathology, The Chinese University of Hong Kong, Prince of Wales Hospital, Hong Kong, China; katiecywong@cuhk.edu.hk; 3 Li Dak Sum Yip Yio Chin R & D Centre for Chinese Medicine, The Chinese University of Hong Kong, Hong Kong, China

**Keywords:** biological pollutants, viruses, bacteria, allergies, chronic inflammation

## Abstract

Environmental pollutants are closely linked to lung cancer. The different types of environmental pollutants can be classified as chemical, physical, and biological. The roles of common chemical and physical pollutants such as PM2.5, smoking, radon, asbestos, and formaldehyde in lung cancer have been extensively studied. Notably, the worldwide COVID-19 pandemic raised awareness of the strong link between biological pollution and human health. Allergens such as house dust mites and pollen, as well as bacteria and viruses, are common biological pollutants. A few biological pollutants have been reported to promote lung cancer via inducing inflammatory cytokines secretion, such as IL-1β, IL-6, and TGF-β, as well as suppressing immunosurveillance by upregulating regulatory T (Treg) cells while dampening the function of CD8^+^ T cells and dendritic cells. However, the correlation between common biological hazards, such as SARS-CoV-2, human immunodeficiency viruses, *Helicobacter pylori*, and house dust mites, and lung cancer is not fully elucidated, and the underlying mechanisms are still unclear. Moreover, the majority of studies that have been performed in lung cancer and biological carcinogens were not based on the perspective of biological pollutants, which has challenged the systematicity and coherence in the field of biological pollutants in lung cancer. Here, in addition to reviewing the recent progress made in investigating the roles of allergens, viruses, and bacteria in lung cancer, we summarized the potential mechanisms underlying biological pollutants in lung cancer. Our narrative review can shed light on understanding the significance of biological pollutants in lung cancer, as well as inspire and broaden research ideas on lung cancer etiology.

## 1. Introduction

Environmental pollution is a well-known risk factor for human cancer [1,2,3,4]. It has been reported that the 1976 industrial tragedy at Seveso, Italy, gradually drew public attention to the influence of environmental pollutants on human health [5]. This accident caused widespread dioxin contamination, leading to a significant increase in cancer rates in the area [6]. Since then, chemical pollutants have become an important topic in the field of environmental pollutants in cancer, whose carcinogenic mechanism is exactly in line with the concept of two-step tumorigenesis: tumor initiation with mutations in healthy cells, followed by a promoter step that triggers cancer development [7]. In addition to chemical pollution, exposures such as tobacco smoke, radon, asbestos, metals, chromium, and arsenic have also been found to be carcinogenic to humans [8]. With the development of science and society, air pollution, including environmental particulate matter ≤2.5 μm (PM2.5), has been identified as an emerging risk factor for cancer, especially lung cancer [9,10]. 

Although lung cancer is currently not the most common type of cancer, it is still the leading cause of cancer mortality worldwide [11]. Lung cancer is frequently utilized to investigate the carcinogenic effects of environmental pollution on humans, since the lung is an important organ exposed to environmental substances and has an essential gas exchange capability for survival. 

It should be emphasized that the global coronavirus disease 19 (COVID-19) pandemic highlighted the perils of biological pollution, demonstrating its substantial detrimental impacts on human health [12]. Alongside chemical and physical pollution, biological pollution is another important part of environmental pollution [13]. The key environmental types of biological pollutants include biological allergens (e.g., cat/dog dander and saliva, house dust mites, cockroaches, molds, and pollen) and microorganisms (viruses and bacteria) [14]. Several studies have shown that pathogenic microorganisms such as severe acute respiratory syndrome coronavirus 2 (SARS-CoV-2) [15,16], human immunodeficiency virus (HIV) [17], human papillomavirus (HPV) [18], Epstein–Barr virus (EBV) [19], *Helicobacter pylori* (*H. pylori*) [20], *Streptococcus pneumoniae* (*S. pneumoniae*) [21], *Mycobacterium tuberculosis* (*M.tb*) [22], *Chlamydia pneumoniae* (*C. pneumoniae*) [23], and the common allergen house dust mite [24] are strongly associated with lung cancer. However, few studies have linked these pathogens/allergens to biological pollutants, and the concept of biological pollutants seems to be partially neglected in research on environmental pollutants and lung cancer, despite the extensive research into air pollution and lung cancer. It is like being in the dark under a lamp when it comes to the state of research on biological pollutants and lung cancer. A lack of systematicity and integrity in research would not only hamper our understanding of the role of biological pollutants in lung cancer, but would also slow the pace of the discovery of new mechanisms and etiologies of lung cancer.

Collectively, this review attempts to summarize the impacts of major biological pollutants on the occurrence and development of lung cancer and their potential mechanisms, which could provide a reference for research on the etiologies and mechanisms of lung cancer. 

## 2. Air Pollution and Lung Cancer

Before reviewing the research on biological pollutants in lung cancer, it is important to have a general understanding of environmental pollution in lung cancer. Bibliometric studies are commonly used in academia to statistically evaluate published research and forecast future trends in scientific inquiry [25]. 

**Figure 1:**
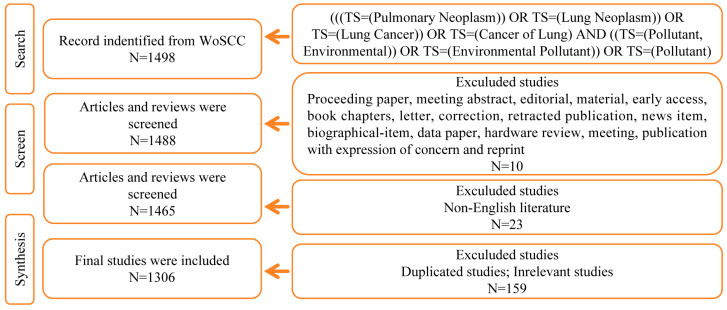
Flowchart depicting the process of publication selection in the field of environmental pollution and lung cancer.

It has been reported that the Web of Science is the “gold standard” for bibliometric use [26]. Therefore, we used the Web of Science Core Collection (WoSCC) database for our bibliometric analysis. We conducted a comprehensive literature search related to environmental pollution and lung cancer within the Science Citation Index-Expanded (SCI-E) of the WoSCC database from 2003 to 2023. The search and reference download were completed within 1 day to prevent bias caused by frequent database updates. We applied the following query to WoSCC: (((TS = (Pulmonary Neoplasm)) OR TS = (Lung Neoplasm)) OR TS = (Lung Cancer)) OR TS = (Cancer of Lung) AND ((TS = (Pollutant, Environmental)) OR TS = (Environmental Pollutant)) OR TS = (Pollutant), with a limited period set from 28 November 2003 to 27 November 2023. The types of documents were limited to articles and reviews, and the language of publication was limited to English. Detailed data retrieval and inclusion procedures are shown in Figure 1. 

**Figure 2:**
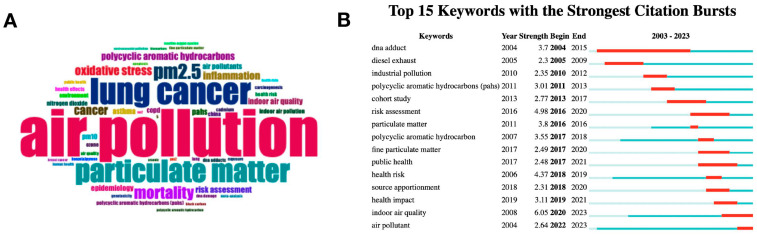
(**A**) Keywords with word cloud generation and (**B**) keywords trend in the field of environmental pollution and lung cancer. Rstudio (version 2023.09.1+494) was used to initiate the online bibliometric analysis tool Bibiometrix. Citespace (6.2.R6) and Bibiometrix were used to analyze the keywords and keyword trends.

**Figure 3:**
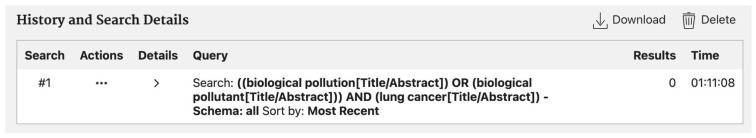
Search results from PubMed on the topics of biological pollution and lung cancer.

Rstudio (version 2023.09.1+494) was used to initiate the online bibliometric analysis tool Bibiometrix. Citespace (6.2.R6) and Bibiometrix were used to analyze the keywords and keyword trends. The word cloud (Figure 2A) showed that air pollution was the most frequent keyword in the fields of environmental pollution and lung cancer over the last 20 years. PM2.5, particulate matter, and polycyclic aromatic hydrocarbons were also the most frequently reported environmental pollutants in lung cancer research. Based on the top 15 keywords with the strongest citation bursts, a clear topic trend in the fields of environmental pollution and lung cancer was shown (Figure 2B). The hot topic in this area has gradually shifted from chemical pollution (e.g., polycyclic aromatic hydrocarbons) to air pollution, with PM2.5 and particulate matter being hotspots in air pollution and lung cancer [27]. However, the effect of biological pollution on lung cancer has not been emphasized in the last 20 years of environmental pollution research based on the results of the bibliometric analysis. Moreover, an advanced research search in PubMed using the query of ((biological pollution (Title/abstract)) OR (biological pollutant (Title/abstract)) AND (lung cancer (Title/abstract)) retrieved zero results on 14 December 2023 (Figure 3), indicating that lung cancer research on the concept of biological pollution is rare.

Therefore, an overview of the research progress in the fields of biological pollution and lung cancer is inevitable. Our review focus on three factors: viruses, common allergens, and bacteria.

## 3. Virus and Lung Cancer

Viruses are strongly associated with lung cancer [28]. SARS-CoV-2 [15,16], HIV [17], HPV [18], and EBV [19] have been attributed as oncogenic viruses and have been shown to play a key role in lung cancer development. However, few publications have systematically and holistically reviewed the research progress on viruses and lung cancer, leading to a fragmented understanding of viruses in lung cancer and an underestimation of the importance of viruses in lung cancer etiology and development. Here, we reviewed the oncogenic viruses that have been reported to show close relationship with lung cancer, and hope to shed light on the investigation in this field. 

### 3.1. SARS-CoV-2

The new coronavirus poses a major threat to the health of all mankind. It has been reported that COVID-19 could increase the risk of lung cancer incidence [16] and promote lung cancer development [29]. A meta-analysis study involving more than 300,000 patients found that COVID-19 increases the mortality rate of lung cancer patients [30]. Research suggests that the SARS-CoV-2 virus, which causes COVID-19, could aggravate lung cancer by inducing chronic inflammation in the lungs [31]. SARS-CoV-2 binds to angiotensin-converting enzyme 2 (ACE-2), which is cleaved by type II transmembrane serine protease (TMPRSS)2 and disintegrin and metallopeptidase domain (ADAM)17 to facilitate its viral entry into host cells [32,33]. SARS-CoV-2 has been reported to promote lung cancer in both direct and indirect ways. On the one hand, SARS-CoV-2 infection promotes cancer cell proliferation and stimulates the progression of the epithelial-to-mesenchymal transition (EMT) of cancer cells by upregulating Zinc-finger E-box-binding homeobox 1 (ZEB1), a well-established regulator of EMT [34]. It has also been speculated that the acute inflammation caused by SARS-CoV-2 may also awaken dormant cancer cells [31]. On the other hand, the dramatic acute inflammation caused by SARS-CoV-2 infection significantly modulates the immune microenvironment in the lung and pulmonary structure [35,36], which consequently deteriorates the development of lung cancer. Multiple inflammation signaling pathways (e.g., nuclear factor-κB (NF-κB) pathway, transforming growth factor beta (TGF-β) pathway, and NLRP3 pathway) are activated, and the cytokine storm induced by SARS-CoV-2 infection forces immune cells (mainly monocyte/macrophage, neutrophil, NK cells, and T cells) to produce cytokines and chemokines, such as tumor necrosis factor α (TNF-α), interleukin (IL)-1β, IL-6, and IL-18, which subsequently enhances the inflammatory condition in the lung tumor microenvironment and leads to cancer development [15,37]. Meanwhile, the exaggerated inflammation induced by SARS-CoV-2 infection alters the lung structure and promotes lung fibrosis by triggering CD163^+^ macrophage responses [36], which further contributes to lung cancer development [38].

### 3.2. HIV

HIV is a risk factor for lung cancer [39]. Lung cancer has been reported to be a leading cause of death in people with HIV [40]. The increased risk of lung cancer in HIV patients is mainly due to smoking, immunosuppression, and inflammatory processes [17]. However, some studies have claimed that the effect of HIV on lung cancer development is independent of the smoking status of patients [39]. Meanwhile, several scientific investigations have been conducted to decipher the mechanisms underlying the increased risk of lung cancer in HIV patients, but this phenotype is not fully understood. CD4^+^ Th cells, the cells primary infected by HIV, have been hypothesized to be correlated with HIV-promoted lung cancer, while several studies have found that the immunosuppression determined by decreased CD4^+^ Th cells only showed a limited association with lung cancer incidence [17]. Nevertheless, a cohort study involving more than 80,000 people reported that HIV patients with a low amount of CD4^+^ Th cells had an increased risk of lung cancer [41]. However, the role of CD4^+^ Th cells in HIV-related lung cancer is still controversial, suggesting that more research is needed on this topic. Furthermore, systemic inflammation associated with HIV infection has also been implicated in lung cancer risk [42]. HIV can trigger pulmonary chronic inflammation by promoting inflammatory cytokine IL-6 secretion [42] and the infiltration of immune cells (CD8^+^ cytotoxic T cells/Tc) [17]. A direct oncogenic role of HIV in lung cancer has also been proposed. An in vitro study showed that the transactivator of transcription protein (Tat) from HIV increased the expression proto-oncogenes, such as c-Myc, c-Fos, and c-Jun, while downregulating the expression of the tumor suppressor gene p53 in a human lung cancer cell line [43]. Interestingly, one study reported that HIV protein infection in immunocompetent mice did not promote lung cancer development, suggesting that host-integrated immunity and components other than HIV proteins are essential for HIV-promoted lung cancer. However, the key component and the major immune response associated with HIV-accelerated lung cancer have not been elucidated. Collectively, chronic inflammation driven by HIV infection could be a potential mechanism underlying HIV-mediated lung cancer, and further investigations in this area are urgently needed to better understand the relationship between HIV infection and increased lung cancer incidence. 

### 3.3. HPV

HPV is a major oncogenic virus, and HPV infection has been reported to increase lung cancer incidence and promote lung cancer development [18,44]. Although the key mechanisms involved in HPV-mediated lung cancer have not been fully elucidated, research advances in this field have shown that HPV can accelerate lung cancer development by promoting EMT, inhibiting cancer cell apoptosis, and exaggerating the inflammation condition in the microenvironment [45]. It has been reported that HPV-infected lung cancer cells exhibited increased proliferative and invasive ability both in vitro and in vivo. Further investigation revealed that Toll-like receptor 3 (TLR3) and epidermal growth factor receptor (EGFR) were the key factors regulating the invasion and EMT capacity of lung cancer, as the inhibition of TLR3 or EGFR expression in cancer cells would significantly attenuate HPV-mediated lung cancer development [45,46]. Moreover, HPV can decrease the expression of p53 in lung tumors and suppress tumor cell apoptosis via upregulation of the inhibitors of apoptosis proteins (IAPs) but downregulation of Bcl-2 homologous antagonist/killer (Bak) [47]. Inflammatory IL-6 and IL-17 secreted by lung cancer cells in response to HPV stimuli would significantly exacerbate the condition of microenvironmental inflammation and lead to lung tumorigenesis and tumor development [48]. 

However, some studies have challenged the idea that HPV infection is a risk factor for lung cancer [49,50,51]. Based on our literature review, we found that one of the major differences between supporting or challenging this idea is the strategy used for the clinical study. Generally, longitudinal and cohort studies were the most common strategies used in clinical studies whose conclusions supported the idea that HPV infection promotes lung cancer [52]. In contrast, the results of cross-sectional studies and case–control studies mainly support the conclusion that HPV infection is not associated with lung cancer [50,51]. Nevertheless, cohort studies appear to be a more appropriate research strategy to elucidate the relationship between HPV infection and lung cancer. In conclusion, further clinical and basic studies on HPV and lung cancer are needed to conclude the association and its underlying mechanisms.

### 3.4. EBV

EBV has been shown to be closely related to lung cancer development [19,53,54]. High levels of EBV DNA in lung cancer patients have been associated with a reduced overall survival (OS) and shorter disease-free survival (DFS) [53]. It is now known that EBV can promote lung cancer by directly altering tumor pathways or by interfering with the expression of immune checkpoint molecules. For example, EBV infection altered the G2/M and G1/S cell cycles in lung cancer cells and affected the activation of the p53, Hippo, and Sirtuin signaling pathways [54], which would promote tumor angiogenesis and cancer proliferation and metastasis. Meanwhile, EBV has been reported to upregulate the expression of immune checkpoint proteins, such as programmed cell death 1 (PD-1), programmed death ligand 1 (PD-L1), cytotoxic T-lymphocyte-associated protein 4 (CTLA-4), and lymphocyte activation gene 3 (LAG3), which strongly facilitate the immuno-evasion capacity of lung cancer [55]. 

## 4. Bacteria and Lung Cancer

Bacteria have long been validated to be associated with lung cancer. Progress in research on human commensal bacteria has revealed that an organ-specific microbiome (e.g., gut, lung, oral, and others) and intra-tumoral bacteria play major roles in lung cancer pathology. However, it is still uncertain if the biological pollutants discussed in this review include the cancer-related microbiome or bacteria, which is a hot topic in recent cancer research. Therefore, a bibliometric analysis cannot be utilized to systematically identify the primary bacteria associated with lung cancer, since it is difficult to determine the keywords employed in the literature search method. It has also been indicated that there is an academic gap between the understanding of biological pollutants, opportunistic pathogens, and pathogenic bacteria, and more investigations and publications are urgently needed to fill this gap. 

Instead, we herein review the research progress made in *H. pylori* [20], *S. pneumoniae* [21], *M.tb* [22], and *C. pneumoniae* [23], which have been reported to be involved in lung cancer development and are not typically recognized as a subject in the research of the human microbiome and lung cancer, although *H. pylori* and *S. pneumoniae* are part of the commensal bacterial flora [56,57]. Consequently, this part of the review aims to inspire people to introduce the concept of biological pollutants in investigations of the microbiome and lung cancer, and to elucidate the relationship between biological hazards and human microbiota. 

### 4.1. H. pylori

*H. pylori* infection has been shown to significantly increase lung cancer risk [20,58,59]. Chronic inflammation promoted by *H. pylori* infection has been suggested to be one of the major pathogenic mechanisms leading to lung cancer. Some studies have shown that infections of *H. pylori* can induce the expression of cyclooxygenase-1 (COX-1), COX-2, and gastrin, which further promotes lung cancer by inducing cell proliferation of the bronchial epithelium [60]. Others believe that *H. pylori* infection promotes the formation of a chronic inflammatory environment by activating the Src kinase (Src) signaling pathway [61,62] and upregulating the expression of pro-inflammatory IL-1, IL-6, and TNF-α [63,64]. Notably, *H. pylori* suppresses the anti-tumor responses of CD8^+^ T cells by altering the antigen-presentation ability of dendritic cells (DCs) [65]. 

### 4.2. S. pneumoniae

*S. pneumoniae* has been reported to play a role in lung cancer development [21,66,67]. Although most studies focus on the effect of *S. pneumoniae* on infections during the treatment of lung cancer patients, limited investigations have, nevertheless, revealed the potential mechanisms underlying *S. pneumoniae*-promoted lung cancer. The extracellular receptors pneumococcal surface protein C (PspC) and platelet-activating factor receptor (PAFR) help *S. pneumoniae* to enter host lung cancer cells, which subsequently activates the phosphoinositide-3-kinase/protein kinase B (PI3K/AKT) and NF-κB signaling pathways, and consequently, accelerates lung cancer progression and metastasis via exaggerating chronic inflammation in the microenvironment [21]. Meanwhile, S. pneumoniae could deteriorate an inflammation condition by upregulating the expression of DNA damage inducible transcript 4 (DDIT4), which further activates the AKT pathway and enhances the migration and invasion of lung cancer cells [67]. However, it is still difficult to conclude that *S. pneumoniae* is a causal factor in the incidence and development of lung cancer due to the rare studies, suggesting that more investigation is needed in this area to fully comprehend the role of *S. pneumoniae* in lung cancer in addition to its infectious characteristics.

### 4.3. M.tb

Pulmonary tuberculosis is a disease caused by *M.tb* infection. *M.tb* has been reported to be a risk factor for lung cancer [68,69]. Pulmonary tuberculosis significantly promotes lung cancer metastasis [70] and increases mortality in lung cancer patients [71]. The PD-1/PD-L1 pathway was found to be involved in *M.tb* infection, which suppress the host Th1 immune response and eventually promotes lung cancer development [72]. Further studies have shown that PD-L1 in combination with the Akt signaling pathway contributed to the increased proportion of CD4^+^Foxp3^+^ regulatory T (Treg) cells in *M.tb* infection, which consequently increased the risk of malignancy by suppressing the host immune response [73]. More importantly, chronic inflammation mediated by *M.tb* infection was found to be the main mechanism bridging pulmonary tuberculosis and lung cancer [22,74]. *M.tb*-induced TNF-α and IL-6 promote the expression of antiapoptotic genes through the NF-κB pathway, which further contributes to the development of lung cancer [74]. Meanwhile, *M.tb* infection leads to an increased release of reactive oxygen species (ROS) [75] and causes mitochondrial DNA (mtDNA) damage. ROS combined with mtDNA damage shorten DNA repair time by suppressing p21 expression, which greatly promotes lung cancer cell division rate [76]. Moreover, M2 macrophages have also been reported to play a major role in *M.tb*-mediated lung cancer. *M.tb* blocks M1 polarization through mediating the transcription of IFN-γ and IL-6, leading to an elevated level of M2 macrophages [77]. M2 macrophages are characterized by high expressions of PD-L1 and CTLA4 [78], which could not only recruit Treg cells via C-C chemokine receptor 4 (CCR4) to exacerbate the immunosuppressive condition in the cancer microenvironment [79], but also secrete anti-apoptotic cytokines such as TGF-β and epidermal growth factor (EGF) to promote tumor cell proliferation [78]. 

### 4.4. C. pneumoniae

*C. pneumoniae* is a Gram-negative bacterium that is a common respiratory pathogen causing chronic and persistent respiratory infections [80]. Studies have shown that *C. pneumoniae* infection significantly increases the risk of lung cancer [23,81,82,83,84]. Although the mechanisms underlying *C. pneumoniae*-promoted lung cancer are still unknown, chronic inflammation caused by *C. pneumoniae* infection is inextricably linked to the development of lung cancer. It has been reported that infections of *C. pneumoniae* trigger the activation of the NF-κB pathway and the expressions of TNF-α, CXCL8 [85], and nitric oxide [86], which would lead to an abnormal inflammatory response and genetic damage. In addition, the upregulation of anti-apoptotic Treg cytokine IL-10 mediated by *C. pneumoniae* infection could block apoptosis, increasing the risk of the vicious transformation of infected cells [87].

### 4.5. Nontypeable Haemophilus influenzae

Nontypeable *Haemophilus influenzae* (NTHi) is a risk factor for chronic obstructive pulmonary disease (COPD), which has been reported to promote lung cancer development in mice in a toll-like receptor (TLR)-dependent manner [88,89]. Further investigation revealed that NTHi promoted K-ras-driven lung cancer via COPD-like airway inflammation, which was mediated by the NF-κB-involved TLR2/4/9 pathway [89]. However, the correlation between NTHi and lung cancer in patients has not been fully elucidated [90], which, in turn, sheds light an opportunity for researchers to fill in this gap. 

## 5. Allergen in Lung Cancer

Many studies have found that allergy and allergic inflammation are closely related to lung cancer [91,92,93,94,95,96,97]. However, the detailed mechanisms underlying allergy-promoting lung cancer are still unclear due to the limited experimental investigations that have been conducted. So far, only one publication has directly demonstrated that chronic exposure to house dust mites (HDMs), a major asthmatic allergen, contributes to lung cancer development [24]. Further studies have shown HDM-promoted lung cancer in a synergetic way. In one way, macrophage activation by HDM exposure triggered the NLRP3-mediated inflammasome pathway. In another way, HDM promoted the expression of monocyte chemoattractant protein-1 (CCL2), which attracted more macrophages into the lung tumor microenvironment. The synergy of these two manners, mediated by long-term HDM exposure, significantly deteriorates the inflammation status in the lung and, consequently, exacerbates lung cancer [24]. 

We also checked the research progress in pollen and lung cancer. To our surprise, though there are a few publications about the correlation between pollen exposure and increased lung cancer incidence [98], the underlying mechanisms have not been well-studied yet. Thus, more clinical and experimental research on allergy and lung cancer is, therefore, urgently needed, which would further help with biomarker selection and the early screening of lung cancer.

## 6. Discussion

As people’s health awareness has increased, the impact of everyday environmental pollution on people’s health has gradually received more and more attention. A close relationship between environmental pollutants and lung cancer has been established, and sufficient evidence has been provided to support a causal link between exposure to environmental pollutants and increased lung cancer incidence and mortality [9]. Environmental pollutants can be further divided into three classes, physical pollutants, chemical pollutants, and biological pollutants. To date, the adverse effects of environmental pollutants on lung cancer have been well established and their potential mechanisms have been extensively studied. However, based on the literature review, we found that most of the research in this area has focused mainly on physical and chemical pollutants, while the harm of biological pollutants on lung cancer has somehow been ignored. However, it should be emphasized that biological pollutants, such as viruses, bacteria, and common allergies, are closely related to human health, including lung cancer, as the COVID-19 pandemic demonstrated. Therefore, our review was based on this research background and aimed to review and summarize the most important research advances in the relationship between biological pollutants and lung cancer. This review could be recognized as a brick to attract jade, which would shed light on the research between biological pollutants and lung cancer and attract more attention in this field.

**Figure 4:**
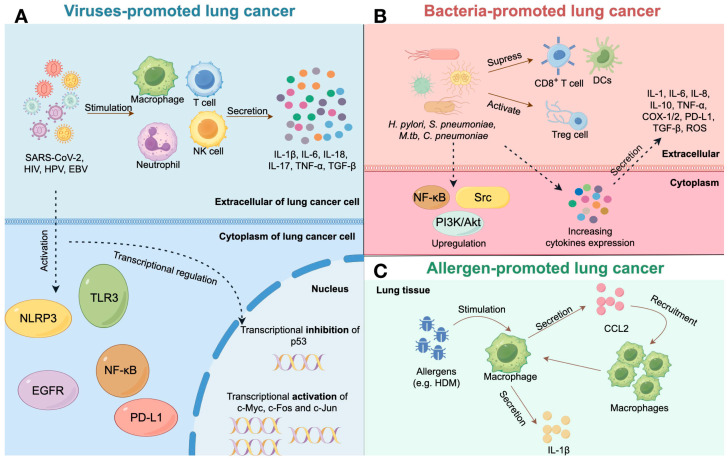
Potential mechanisms underlying biological-pollutant-promoted lung cancer. Viruses, bacteria, and allergens are major types of biological pollutants and their possible pathogenic mechanisms on lung cancer are summarized based on the latest research progress. (**A**) SARS-CoV-2, HIV, HPV, and EBV can promote lung cancer development in two ways: by increasing pro-inflammatory cytokine expression in immune cells, or by directly activating inflammation signaling pathways in infected lung cancer cells, combined with up-regulation of oncogene transcription and downregulation of tumor suppressor gene. (**B**) *H. pylori*, *S. pneumoniae*, *M.tb*, and *C. pneumoniae* could increase lung cancer risk via dysfunction of CD8^+^ Tc cells and DCs, as well as promoting Treg cell proliferation. These bacteria can also stimulate inflammation signaling and promote inflammatory cytokine expression in the host. (**C**) Experimental research on allergens and lung cancer is rare, while a common asthmatic allergen HDM could promote lung cancer development via triggering macrophage-mediated NRLP3 activation and IL-1β expression.

The limitations of this review, due to the paucity of publications, are twofold: the unclear association between specific biological pollutants and lung cancer, and the mechanisms that have not been fully elucidated. In the case of viruses, HCV has been reported to play a role in lung tumorigenesis [99,100]. Some studies have suggested that HCV could inhibit cancer cell apoptosis by interfering with the p53 signaling pathway and downregulating pro-apoptotic proteins, such as caspase-3 and TNF-α [101,102]. In addition, HCV has been shown to inhibit the proliferation of NK cells and further suppress the secretion of cytokines and cytotoxic granules [103]. However, whether HPV is a risk factor for lung cancer is still elusive due to the scarce investigations and their conflicting results. Some clinical studies have not found a significant correlation between HPV infection and increased lung cancer incidence [104,105]. The same issue occurs with research on HBV and lung cancer, and previous findings have shown that HBV infection has a limited role in lung cancer risk [106]. Furthermore, the mechanisms that have been reported to explain the relationship between bio-contaminant exposure and increased lung cancer incidence and stage can all be attributed to the involvement of chronic inflammation, which, to some extent, reveals the adequate condition of mechanistic studies, but also suggests that chronic inflammation may be the ultimate mechanism explaining the pathogenesis of lung cancer (Figure 4).

## 7. Conclusions

In conclusion, exposure to major biological pollutants such as viruses, pathogenic bacteria, and allergies can exaggerate host inflammatory condition and lead to chronic inflammation, which could contribute to increased lung cancer incidence and malignancy. More research on this topic is urgently needed, which would not only benefit the etiological investigation of lung cancer, but also contribute to novel drug development based on new scientific findings. 

## Data Availability

No new research data to be shared in this review.

## Abbreviations

ACE-2, angiotensin-converting enzyme 2; ADAM, disintegrin and metallopeptidase domain; AKT, protein kinase B; Bak, Bcl-2 homologous antagonist/killer; CCL2, monocyte chemoattractant protein-1; CCR4, C-C chemokine receptor 4; COVID-19, coronavirus disease 19; COX-1, cyclooxygenase-1; COX-2, cyclooxygenase-2; DCs, dendritic cells; DDIT4, DNA damage inducible transcript 4; DFS, disease-free survival; EBV, Epstein–Barr virus; EGF, epidermal growth factor; EGFR, epidermal growth factor receptor; EMT, epithelial-to-mesenchymal transition; H. pylori, Helicobacter pylori; HDM, house dust mite; HIV, human immunodeficiency viruses; HPV, human papillomavirus; IAPs, inhibitors of antiapoptosis proteins; IL-1β, interleukin-1β; M.tb, Mycobacterium tuberculosis; mtDNA, mitochondrial DNA; NF-κB, nuclear factor κB; OS, overall survival; PAFR, platelet-activating factor receptor; PI3K, phosphoinositide-3-kinase; PspC, pneumococcal surface protein C; ROS, reactive oxygen species; S. pneumoniae, Streptococcus pneumoniae; SARS-CoV-2, syndrome coronavirus 2; Src, Src kinase; Tat, transactivator of transcription protein; TGF-β, transforming growth factor beta; TLR3, Toll-like receptor 3; TMPRSS2, type II transmembrane serine protease; TNF-α, tumor necrosis factor α; Treg cells, regulatory T cells; ZEB1, Zinc-finger E-box-binding homeobox 1.
